# Possible niche compression and individual specialization in Pacific Arctic beluga (*Delphinapterus leucas*) from the 19th to 20th century

**DOI:** 10.1002/ece3.10230

**Published:** 2023-07-04

**Authors:** Devin C. Fraleigh, Frederick I. Archer, Amanda S. Williard, Luis A. Hückstädt, Alyson H. Fleming

**Affiliations:** ^1^ Center for Marine Science University of North Carolina Wilmington Wilmington North Carolina USA; ^2^ Southwest Fisheries Science Center National Marine Fisheries Service, National Oceanic and Atmospheric Administration La Jolla California USA; ^3^ Department of Biology and Marine Biology University of North Carolina Wilmington Wilmington North Carolina USA; ^4^ Centre for Ecology and Conservation University of Exeter Cornwall UK; ^5^ Department of Forest & Wildlife Ecology University of Wisconsin‐Madison Madison Wisconsin USA; ^6^ National Museum of Natural History Smithsonian Institution Washington District of Columbia USA

**Keywords:** beluga, growth layer groups, isotope, niche, Pacific Arctic, specialization

## Abstract

Cetaceans have shown a potential to be used as sentinel species for tracking environmental change in marine ecosystems, yet our assessment of change is typically limited to recent decades and lacks ecological baselines. Using historical museum specimens, we compared community niche metrics and degree of individual dietary specialization in groups of Pacific Arctic beluga (*Delphinapterus leucas*) from the 1800s (*n* = 5) to 1900s (*n* = 10) using stable carbon and nitrogen isotopes drilled from teeth. Beluga occupied a broader trophic niche and demonstrated a higher degree of individual specialization in the 1800s than in the 1900s. The cause of this shift is difficult to confirm given long timescales and constraints of specimen‐based research but could indicate changes in the prey base or competition. The scale and nature of this detected shift provide perspective for continued research on these climate‐vulnerable species.

## Graphical abstract

Community niche metrics and dietary specialization at the individual level were assessed between beluga whales from the 1800s to 1900s using carbon and nitrogen‐stable isotopes from museum teeth specimens. Population niche width of sampled beluga from the 1800s was greater than that of the 1900s, and individuals in the 1800s occupied a smaller area relative to their total available niche, indicating a higher degree of individual specialization than 1900s beluga. However, individual beluga niche widths were similar between centuries.

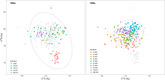

## 1 INTRODUCTION

Globally the marine environment has been undergoing rapid change due to anthropogenic activities (Field et al., 2014; Gattuso et al., 2018). This is especially true for the high‐latitude oceans, where warming is occurring faster than the rest of the world (Blunden & Arndt, 2016; Neukermans et al., 2018). In the Pacific Arctic, changing environmental conditions have been documented in the area where the North Pacific meets the Arctic Ocean encompassing the Bering, Beaufort, and Chukchi Seas, including dramatic reductions in sea ice extent (Grebmeier et al., 2010), shifts in biological communities (Brodeur et al., 2008; Will et al., 2020), changes in patterns of primary production (Arrigo & van Dijken, 2015), and alterations in species phenology (e.g., timing of phytoplankton blooms, ice dynamics, and species migrations and reproduction; Hill et al., 2018; Moore, 2016). How species will fare in the Pacific Arctic during this period of environmental change and human pressures (e.g, shipping, hunting, overfishing) will depend on a multitude of factors including not only the type, magnitude, and persistence of these stressors but also the biology, ecology, and behavioral responses displayed by species at the individual and population levels.

Beluga whales (*Delphinapterus leucas*) are a particularly valuable sentinel species for understanding the magnitude and impact of environmental change in the Arctic and sub‐Arctic due to their long lives, circum‐Arctic distribution, and association with sea ice (Stewart & Stewart, 1989). Additionally, they are both culturally and nutritionally important for Arctic indigenous groups. Multiple populations currently inhabit the Pacific Arctic and include the (1) Eastern Chukchi Sea, (2) Beaufort Sea, (3) Bristol Bay, (4) Cook Inlet, (5) Eastern Bering Sea, and (6) Anadyr populations (Nelson et al., 2018; O'Corry‐Crowe et al., 2018). As a species, beluga have a generalist diet, with whales consuming a wide variety of prey including fish, cephalopods, and invertebrates, although Arctic cod (*Boreogadis saida*) is perhaps the most important prey item, especially for northern populations (Loseto et al., 2009; Quakenbush et al., 2015). The degree of individual dietary specialization in beluga is not well understood, and certain populations likely exhibit more dietary specialization than others. Variation in foraging behaviors has been documented between and even within populations depending on age and sex (Marcoux et al., 2012; Quakenbush et al., 2015).

There is currently a large degree of uncertainty as to how climate change and warming oceans are affecting beluga populations in the Pacific Arctic and how they will continue to affect beluga in the coming decades. Already, multiple pieces of evidence are suggesting that these whales are exhibiting signs of response to environmental change, including a decline in growth rates, alterations of migration routes and/or timing, and dietary switches (Bailleul et al., 2012; Choy et al., 2020; Harwood et al., 2015; Hauser et al., 2017; O'Corry‐Crowe et al., 2016). Understanding how beluga have responded to changing conditions in the past is likely to improve predictions of how their populations in the Pacific Arctic might respond to current and future environmental changes. Museum specimens, including bones, teeth, hair, feathers, and baleen can be used to examine past ecosystem conditions and species behavior. Beluga teeth grow continuously over the course of an individual's life and are composed of identifiable growth layer groups (GLGs), with approximately one GLG being deposited each year (Luque et al., 2007; Nelson et al., 2018; Waugh et al., 2018). These GLGs are formed in metabolically inert dentin which remain unchanged once deposited, so a single tooth can be sequentially sampled to provide a plethora of data points over the course of a beluga's life with a temporal resolution of 1 year. Other studies have used sequential sampling of metabolically inert tissues over continuous timescales in arctic marine mammals, including beluga, narwhals, bowhead whales, ringed seals, and harp seals (Boucher et al., 2020; Dietz et al., 2021; Kershaw et al., 2021; Nelson et al., 2018; Schell, 2000).

Stable isotope analysis (SIA) has proven to be an invaluable tool for studying the biology, ecology, and physiology of living organisms, including difficult‐to‐study marine mammals (Fleming et al., 2018; Fry, 2006; Newsome, Martinez del Rio, et al., 2007; Smith et al., 2022; Wolf et al., 2009) as ratios of isotopes behave, at least to some degree, in predictable ways based on life history traits, environmental conditions, and species physiology. Stable isotope ratios of carbon (^13^C/^12^C) and nitrogen (^15^N/^14^N) are particularly informative for foraging ecology, as carbon is indicative of patterns of primary production and therefore foraging habitat (e.g., coastal and benthic areas are enriched in δ^13^C over offshore and pelagic areas), and nitrogen is reflective of the trophic level of consumer species and nitrogen processes at the base of the food web (Ben‐David & Flaherty, 2012; Kurle & McWhorter, 2017). Patterns of δ^13^C in the ocean are dictated by primary producer community structure and growth dynamics, resource availability, and sources of dissolved inorganic carbon (DIC) as well as by terrestrial input (e.g., rivers) and, during the last century, the burning of fossil fuels by humans (known as the Suess effect; Barnes et al., 2009; Gradinger, 2009; Vega et al., 2019). δ^15^N trends are driven by the availability and dominance of certain nitrogen species in seawater, where higher levels of denitrification will lead to more enriched δ^15^N ratios than areas with higher levels of nitrogen fixation (Cline & Kaplan, 1975; Kurle & McWhorter, 2017). Additionally, and of particular importance for dietary studies, nitrogen undergoes substantial fractionation during metabolic processes, leaving tissues of a consumer more enriched in the heavier ^15^N isotopes with each increase in trophic level (Brault et al., 2019; DeNiro & Epstein, 1981; Minagawa & Wada, 1984). Isotope ratios in a consumer's tissues are reflective of diet during the period of time in which the tissue was synthesized; in this sense, δ^13^C and δ^15^N from beluga GLGs represent yearly averages of dietary information for an individual. In addition, as both δ^13^C and δ^15^N are driven by an animal's habitat and resource utilization, together these ratios can be used as a two‐dimensional representation of the *n*‐dimensional niche which an individual occupies (Hutchinson, 1957; Newsome, Martinez del Rio, et al., 2007).

Many species of marine mammals exhibit high degrees of behavioral plasticity and individuality in how they occupy their ecological niche (Fleming et al., 2016; Hutchinson, 1957; Ramp et al., 2015; Samarra & Miller, 2015; Schwarz et al., 2021). A combination of pressures exerted on a consumer (e.g., intra or interspecific competition, predation, prey availability), their behavioral response to them, and other extrinsic environmental factors all act to shape the realized trophic niche at both individual and population levels (Bolnick et al., 2010; Hayden et al., 2019). For example, strong intraspecific competition among a predator species might be expected to result in a greater niche width at the population level and a higher degree of trophic variability (or dissimilarity) between individuals of the population (Bolnick et al., 2010).

A set of community niche metrics have been developed to use stable isotope ratios to quantify food web characteristics, describing the niche widths of communities as a whole as well as how groups of taxa are packed within that overall niche in carbon–nitrogen isospace (Layman, Arrington, et al., 2007). These metrics have been used for studies examining how trophic niches are affected by habitat fragmentation, novel species invasions, and man‐made ecological disasters, among others (Andrades et al., 2020; Jackson et al., 2012; Layman, Quattrochi, et al., 2007). Yet these same metrics also lend themselves well to studies of high trophic level consumers, and when applied to beluga are indicative of the occupied niche width at the population level and individual dietary specialization (Layman, Arrington, et al., 2007).

The degree of dietary specialization of an individual can be calculated as the niche occupied by an individual when compared to the total niche width available to the individual (defined here as the niche occupied by the population as a whole; Bearhop et al., 2004; Bolnick et al., 2002). An individual is considered to have a specialist diet when its realized niche width is relatively small compared to its total available niche width, while an individual occupying a larger niche width relative to its total available niche width would be considered to have a more generalist diet (Araújo et al., 2007; Newsome et al., 2009). This conceptualization of individual dietary specialization has been applied to a number of taxa, including southern sea otters (*Enhydra lutris nereis*), southern elephant seals (*Mirounga leonina*), bull sharks (*Carcharhinus leucas*), tiger sharks (*Galeocerdo cuvier*), and leptodactylid frogs, by using stable isotope ratios of δ^13^C and δ^15^N taken from tissues (Araújo et al., 2007; Hückstädt et al., 2012; Matich et al., 2011; Newsome et al., 2009). The degree of individual dietary specialization within a species can have important ramifications regarding how that species might persist in the face of environmental change and other anthropogenic stressors.

In this paper, we aim to describe beluga foraging ecology in the Pacific Arctic from the 1800s to the 1900s using δ^13^C and δ^15^N values from individual tooth GLGs in an attempt to understand how populations may have changed in the past and consequently how they may continue to adapt in an uncertain future. Specifically, our goals are to (1) compare community isotopic niche metrics between beluga of both centuries and (2) determine the level of individual specialization in beluga by comparing the within‐individual isotope variability from samples taken from each GLG to the within‐century variation across all individuals. This study will help place modern studies of beluga into context by providing historical baseline isotope data and may allow for better‐informed management of the system and species in the future.

## 2 METHODS

### 2.1 Sample collection

**FIGURE 1:**
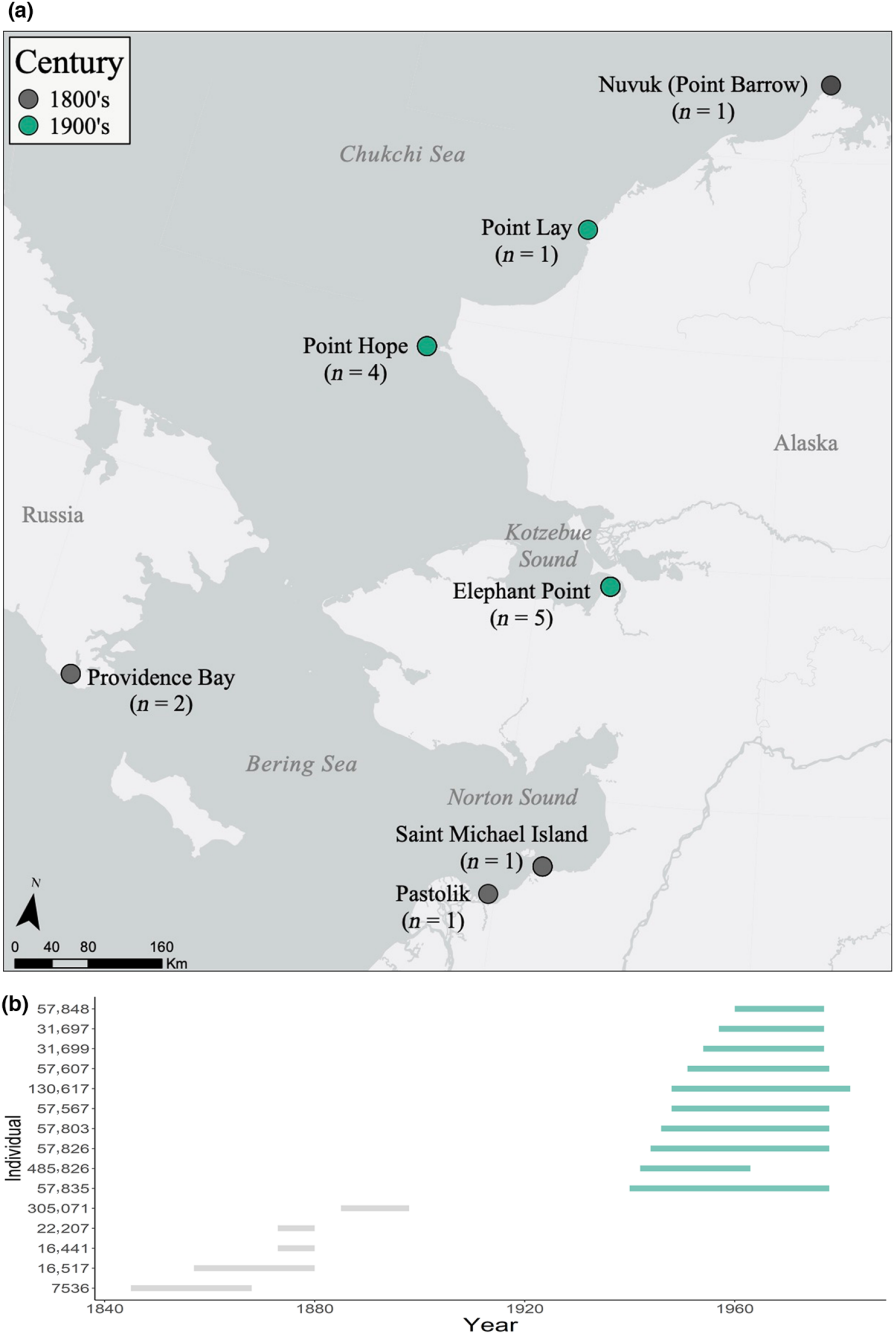
(a) Map of the study area and collection locations of teeth. Gray circles represent teeth from the 1800s and green circles represent teeth from the 1900s. (b) Temporal coverage of each individual beluga.

To ensure a broad temporal coverage of beluga tooth isotope data from the Pacific Arctic, teeth were sourced from multiple locations throughout the Pacific Arctic and from two distinct time periods. Teeth with GLGs ranging in date from the latter half of the 19th century (approximately 1845–1898; *n* = 5) and the mid‐20th century (1940–1983; *n* = 10) were loaned with permission for destructive sampling from the Smithsonian National Museum of Natural History in Washington, DC and the University of Alaska's Museum of the North (UAM) in Fairbanks, Alaska. Specific localities of harvested, captured, or found beluga, as well as temporal coverage of data from these individuals, can be found in Figure 1 and Table 1. Specifics regarding original collection methods are unknown, although it is very likely that 1800s samples came from early scientific expeditions and 1900s samples were sourced from indigenous harvests of whales during migrations. While localities were included for all the specimens, further details on population assignment were unfortunately not available. Sex was unknown for all but one beluga from the 1800s; a young female which was 8 years old at death. Out of the 10 whales from the 1900s, there were four males, four females, and two individuals of unknown sex.

**TABLE 1:** Metadata for all 15 belugas included in this study. Catalog numbers are as listed in the respective museum (USNM, Smithsonian Institution's National Museum of Natural History & UAM, University of Alaska Museum of the North). Samples drilled do not necessarily match the number of years of coverage, as some GLGs were combined together into one sample.

Museum	Catalog number	Start year	End year	Samples drilled	Age at death	Precise locality	Sex
USNM	A 7536	1845^a^	1868^a^	24	24	Pastolik	Unknown
USNM	A 16517	1857	1880	24	24	Plover Bay	Unknown
USNM	A 16441	1873	1880	8	8	Plover Bay	Unknown
USNM	A 22207	1873	1880	8	8	Saint Michael Island	Female
USNM	305071	1885	1898	14	14	Point Barrow	Unknown
UAM	57835	1940	1978	28	39	Elephant Point	Male
USNM	485826	1942	1963	22	22	Elephant Point	Unknown
UAM	57826	1944	1978	27	35	Elephant Point	Male
UAM	57803	1946	1978	24	33	Elephant Point	Female
UAM	57567	1948	1978	26	31	Elephant Point	Female
UAM	130617	1948	1983	26	36	Point Hope	Male
UAM	57607	1951	1978	25	28	Point Lay	Unknown
UAM	31699	1954	1977	14	24	Point Hope	Female
UAM	31697	1957	1977	17	21	Point Hope	Female
UAM	57848	1959	1977	17	20	Point Hope	Male

^a^ Note that the individual with a catalog number of 7536 was not archived with a precise collection year; we assumed a death date of 1868 due to the collector, William Healey Dall, being in the Norton Sound near Pastolik at this time (Woodring, 1958).

### 2.2 Stable isotope analysis

Upon receipt from museum collections, teeth were cut in half longitudinally to reveal GLGs. Powder was drilled from every GLG when possible, providing a temporal resolution of 1 year. For some teeth with extremely narrow GLGs two, three, or in one single case four layers were combined, resulting in a total of 304 drilled samples. When multiple GLGs were combined, a “mean year” value was given for that sample by taking the mean of included years and rounding up to the nearest year. Drilling was done with either a computer‐guided micromilling system (Carpenter Microsystems CM‐2, version 3.0.6, Iowa City, IA, USA) with an NSK Volvere Vmax drill, fitted with a 010‐mm carbide, round‐tipped bit (model H71.11.004 by Brasseler USA Dental Instruments) or a handheld Dremel tool (model 2050) with the same round‐tipped bit. Samples were weighed (target 1.5 mg), placed in tin cups, and run for bulk stable isotope analysis of δ^13^C and δ^15^N values at the University of California Santa Cruz (UCSC) Stable Isotope Laboratory (SIL; Smithsonian samples) and the IRMS lab at the Center for Marine Science at the University of North Carolina Wilmington, UNCW (UAM samples). Samples run at UCSC were analyzed using a Carbo Elba NC2500 elemental analyzer coupled with a Thermo Scientific Delta Plus XP isotope ratio mass spectrometer, whereas samples run at UNCW were analyzed via a Costech 4010 elemental analyzer coupled with a Thermo Scientific Delta‐V Plus isotope ratio mass spectrometer. It is important to note there is the possibility for some variation between the two facilities, yet measures were taken to ensure data were as accurate as possible. Within run quality control and mass drift corrections were assessed using glutamic acid (USGS 40 & USGS 41a; UNCW) and a gelatin standard reference material (PUGel; UCSC). All carbon isotope values were corrected for the Suess and Laws effects to the year 1850 using the SuessR package (Clark et al., 2021), with “region” set to the Bering Sea. For the few GLGs that were dated prior to 1850, no corrections were made. All isotopic values are reported in reference to their accepted standards, Vienna PeeDee Belemnite (V‐PDB) for δ^13^C and air (N_2_) for δ^15^N.

### 2.3 Statistical analyses

All statistical analyses were conducted using R (version 4.0.2; R Core Team, 2020). Wilcoxon tests were used to compare δ^13^C and δ^15^N between males and females from the 1900s beluga, with mean values per individual (*n* = 4 females and *n* = 4 males). Of the 1800s beluga, only one had known sex, a female, so no analyses were made for individuals in this group. Due to the opportunistic nature of acquiring museum specimens for research, individual beluga were harvested over a large geographic area with differential spatial and temporal coverage across the two time periods (1800s and 1900s; see Figure 1). Given this uneven sampling across centuries and the nature of isotope signatures, particularly carbon, at the base of the food web to vary predictably through space and time (Goericke & Fry, 1994; Kurle & McWhorter, 2017), we first wanted to explore the potential scope and relative influence of both location and year on the beluga isotope signatures. A pairwise comparison of all sampled points was made, where isotope ratios of every single GLG were compared with every other GLG in the dataset. This resulted in a new dataset with the change of δ^13^C and δ^15^N (∆ δ^13^C and ∆ δ^15^N) between each pair, as well as the geographic distance between paired samples (zero for GLGs from the same whale or multiple whales taken from the same location) calculated using the package *swfscMisc* (Archer, 2022) and the difference in time (years) between paired samples. The effect that geographic distance and difference in time had on the change in δ^13^C or δ^15^N was addressed using linear regressions.

Community niche metrics, hereafter referred to as Layman metrics, were calculated using the package SIBER (Jackson et al., 2011; Layman, Arrington, et al., 2007). All six of the following metrics were calculated per model iteration, and the modes and 95% credible intervals, the latter of which were calculated as the highest density region, of the resulting posteriors are reported here. Total area (TA) represents the minimum area in isospace (the two‐dimensional space with δ^13^C as the *x*‐axis and δ^15^N as the *y*‐axis) which a polygon occupies while including centroid locations for each individual of a community (in this case century). Range in δ^13^C (CR) and δ^15^N (NR) is the one‐dimensional difference between maximum and minimum values for each isotopic ratio and is similar to TA in that it is reflective of trophic niche width in a community. The mean distance to the centroid (CD) is the mean distance of each individual beluga to the mean of all beluga in the given community (century). Last, the mean distance to the nearest neighbor (MNND) and standard deviation distance to the nearest neighbor (SDNND) are the mean and standard deviation, respectively, of each beluga to its nearest neighbor in isospace. Bayesian models with vague priors were fitted to each individual beluga to calculate these metrics as well as standard ellipse areas (SEA) at the individual level. Each model consisted of five MCMC chains of 1,000,000 iterations with a thinning rate of 100. Model convergence was assessed by checking Gelman‐Rubin diagnostics and assurance was made toward each chain having low autocorrelation. Note that all Layman metrics were calculated per model iteration and that these metrics, as well as SEA, are reported here as modes and 95% credible intervals of the posterior draws. To describe differences in isotope ratios between the two centuries, a new posterior of the difference in δ^13^C and δ^15^N over time was calculated by taking the mean δ^13^C and δ^15^N per posterior draw of each individual for each century and subtracting the 1900s means from the 1800s means (creating a list of the same length as the original number of posterior draws).

To calculate the degree of individual specialization in beluga the following formula was used: *S* = WIC/(BIC + WIC), where *S* is the specialization index, WIC is the within‐individual component, BIC is the between‐individual component, and the sum of BIC and WIC is the total niche width of the population (Bearhop et al., 2004; Hückstädt et al., 2012; Newsome et al., 2009). The smaller the value of *S*, the more specialized an individual is considered in relation to the total niche width available to that individual. Previous work has considered an *S* ≤ 0.2 to be an extreme specialist whereas an *S* ≥ 0.5 would indicate a generalist individual (Hückstädt et al., 2012). The WIC and BIC are calculated as the standard deviation of a set of points—here the WIC is the standard deviation of all sampled GLGs in an individual whale and the BIC is the standard deviation of all sampled GLGs of all beluga in the respective time period (1800s vs 1900s). It is important to note that the degree of individual specialization can be affected not only by changing an individual's WIC, but also by an expansion or contraction of the population's total niche width.

## 3 RESULTS

**FIGURE 2:**
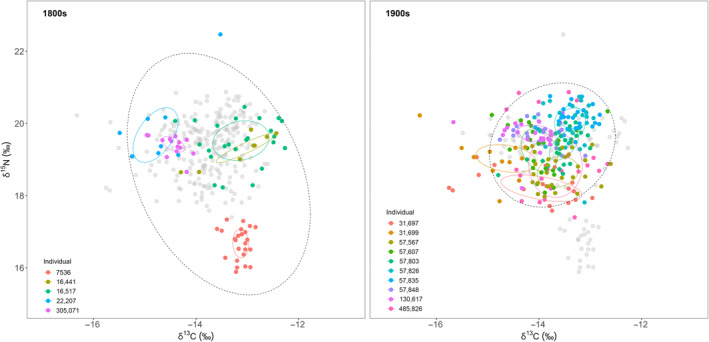
Isospace biplot of all beluga broken down by century. Black dotted ellipses represent approximately 95% of all points of a century, while colored solid ellipses represent approximately 40% of data for each individual beluga. Grey, semi‐transparent points represent whales from the other century for a visual aid. Within individual component (WIC) remained similar between groups while between individual component (BIC) decreased in the 1900s, resulting in a higher degree of generalization of diet relative to total niche width.

Mean δ^13^C for all beluga was −13.72‰ and mean δ^15^N was 19.18‰ (Figure 2). When broken down by century, 1800s beluga had a mean δ^13^C of −13.54‰ and a mean δ^15^N of 18.63‰. Beluga from the 1900s had a mean δ^13^C of −13.78‰ and a mean δ^15^N of 19.36‰. There were no significant differences between 1900s females and males for δ^13^C (*p* = .471), but δ^15^N was greater in males than females (*p* = .030). Mean δ^13^C for the four included females was −13.88‰ and for the four included males was −13.68‰. Mean δ^15^N for females was 18.84‰ and for males was 19.89‰. Results of the Bayesian models and the calculation of a new posterior by subtracting 1900s beluga predicted isotope ratios from 1800s beluga revealed some distinction between centuries for both ratios; for δ^13^C the mode of 1800s–1900s δ^13^C and δ^15^N was 0.129‰ (95% credible interval −0.184‰ to 0.441‰) and −0.350‰ (−0.669‰ to −0.039‰) respectively. The probability that δ^13^C and δ^15^N from 1800s beluga were greater than that of 1900 beluga, calculated as the percent of the posterior ≥0 was 79.5% for δ^13^C and 1.4% for δ^15^N. Conversely, the probability that 1800s δ^13^C and δ^15^N was lower than those from the 1900s was 20.5% and 98.6%, respectively.

### 3.1 Pairwise comparison analysis

With a total of 304 sampled GLGs, the pairwise comparison resulted in a set of 46,056 paired observations. Linear models for both δ^13^C and δ^15^N showed a statistically significant relationship between geographic distance and time between samples and resulting isotope ratios (*p* < .001). However, the models estimated that on average, 1 km is related to a change in δ^13^C of 0.0007‰ and a change in δ^15^N of −0.0003‰/km. The spatial scope of our samples' collection locations extends approximately 880 km on a north–south axis and 770 km east–west. This corresponds to an approximate change of δ^13^C of 0.62‰ latitudinally and 0.54‰ longitudinally, and a change in δ^15^N of −0.26‰ latitudinally and −0.23‰ longitudinally across the study area. Temporally, the models predicted an annual average change of −0.0012‰/yr for δ^13^C and 0.0084‰/yr for δ^15^N. When applied to the temporal scope of our samples, this is a change in δ^13^C of approximately −0.17‰ and a change in δ^15^N of approximately 1.2‰ over the 138‐year time period. The models also estimated that the average difference among individuals sampled in the same year and location (the intercept) is approximately 0.56‰ for δ^13^C and 0.88‰ for δ^15^N. Thus, differences in δ^13^C are greater over distance and δ^15^N changes more over time, as expected, yet both vary by levels comparable to differences among individuals sampled in the same year and location, suggesting neither variable on its own is predictive in a linear manner nor biologically meaningful in the differences observed across the beluga GLG results.

### 3.2 Layman metrics and standard ellipse areas

**FIGURE 3:**
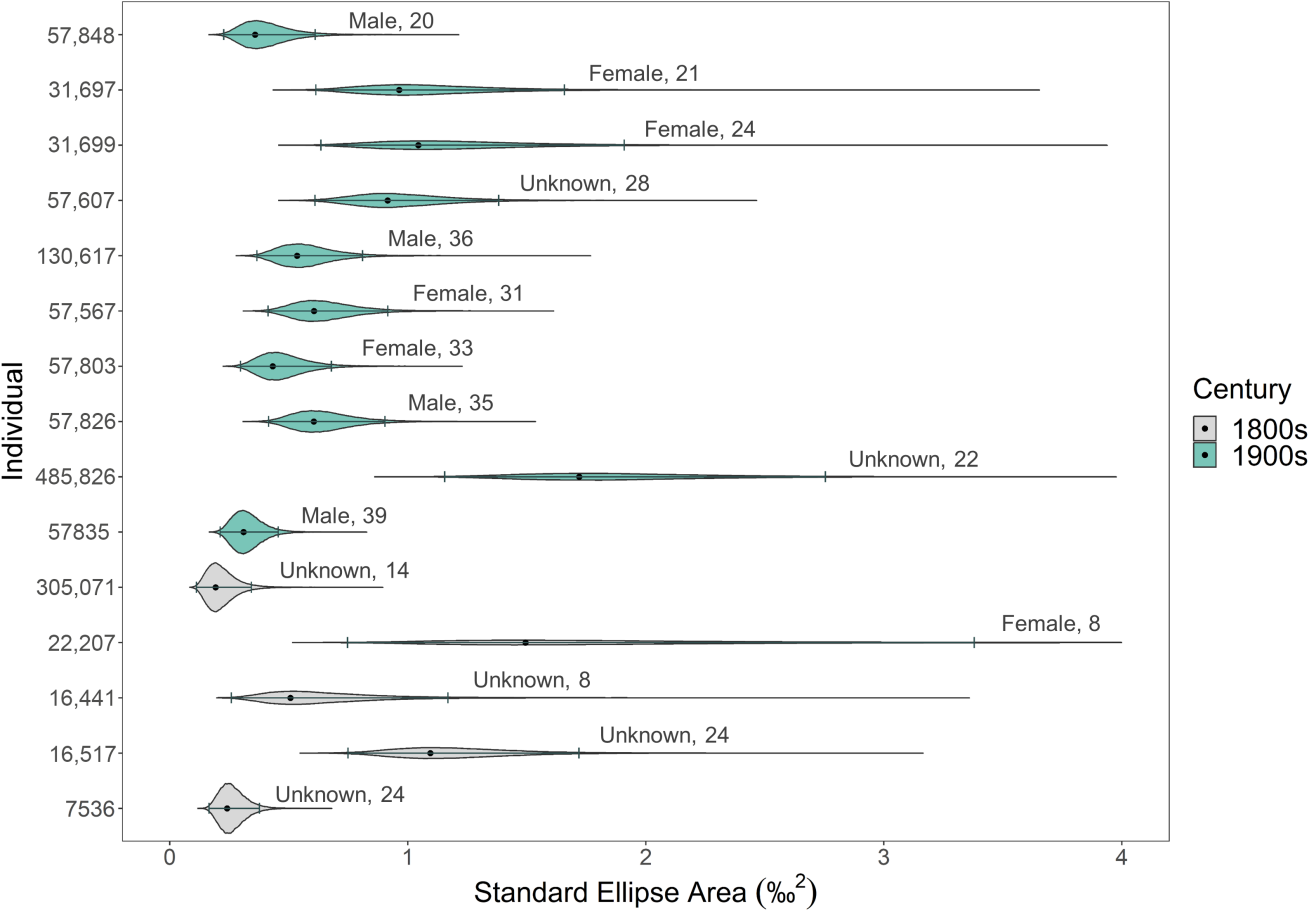
Standard ellipse areas were calculated for each individual beluga from Bayesian models in SIBER. Violins are made from posterior draws, and dots and error bars are modes and 95% credible intervals, respectively. Values are in ‰^2^ and color corresponds to century. Individuals are sorted based on the year of earliest temporal coverage, with the date increasing up the *y* axis. Sex and age at death for each individual are included with violins.

Gelman‐Rubin diagnostics for each model indicated strong convergence of MCMC chains and confidence in the resulting posterior draws. Additionally, autocorrelation along chains was found to be low (≤0.01) when checked at lags of 100, 500, 1000, and 5000. All six Layman metrics were greater for 1800s beluga than 1900s beluga. Modes and Bayesian credible intervals are reported in Table 2. The greatest discrepancies between the two centuries were found in TA (the total area in carbon–nitrogen isospace) and the range in δ^15^N, the latter of which contributed more to TA than did the range in δ^13^C. Standard ellipse areas (hereafter SEA) for each individual beluga were plotted in δ^13^C–δ^15^N isospace (in ‰^2^; Figure 3). 1800s beluga had similar SEA to 1900s beluga (Figure 3). SEA ranged from a low of 0.19‰^2^ for individual 305,071 from Nuvuk (Point Barrow; 1800s) to a high of 1.73‰^2^ for individual 485,826 from Kotzebue Sound (1900s).

**TABLE 2:** Layman metrics calculated from Bayesian models in SIBER for beluga when pooled together by century. Modes and Bayesian 95% credible intervals are reported.

Metric	1800s	1900s
Nitrogen range	3.230 (2.573–4.088)	1.956 (1.420–2.543)
Carbon range	1.802 (1.129–2.700)	1.428 (0.911–2.071)
Total area	2.652 (1.511–4.435)	1.551 (0.901–2.391)
CD	1.229 (1.006–1.490)	0.658 (0.537–0.792)
MNND	1.028 (0.699–1.346)	0.383 (0.257–0.508)
SDNND	0.928 (0.494–1.233)	0.211 (0.085–0.355)

Abbreviations: CD, mean distance to centroid; MNND, mean distance to nearest neighbor; SDNND, standard deviation distance to nearest neighbor.

### 3.3 Individual specialization

**FIGURE 4:**
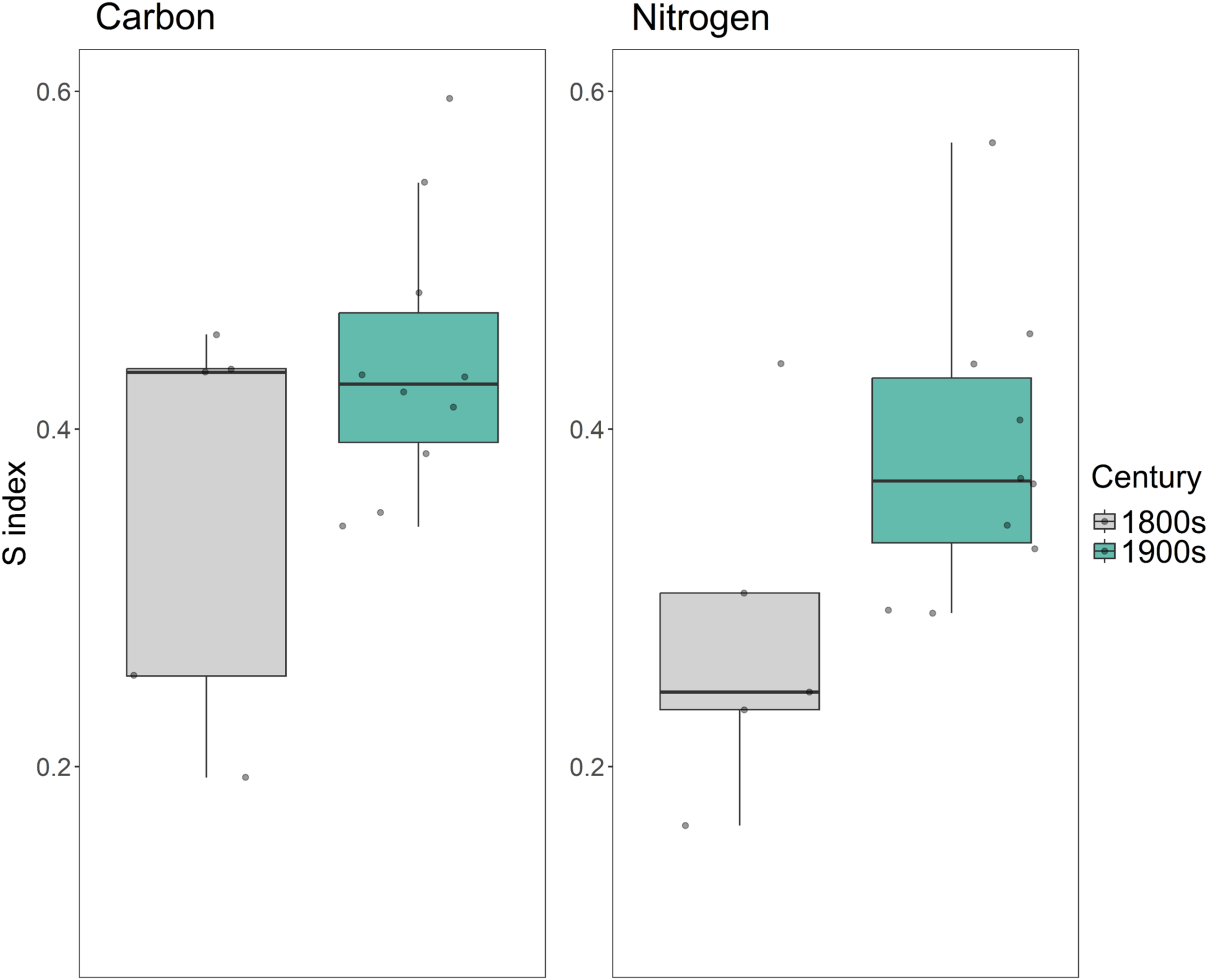
Individual specialization index calculated from carbon and nitrogen ratios. The lower the value, the more specialized the individual whale. Overall, individual beluga appear to have utilized more of their total available resource pool during the 1900s as the S index, and level of generalization of diet, increased.

The degree of individual specialization in Pacific Arctic beluga, defined as each individual's observed occupied niche compared to the population's total available niche, was on average greater in the 1800s than in the 1900s (Table 3, Figure 4). The *S* index, where lower values indicate a higher degree of individual specialization, was lower for both δ^13^C and δ^15^N in the 1800s. Mean ± SD values of *S* for whales from the 1800s were 0.355 ± 0.122 for carbon and 0.277 ± 0.103 for nitrogen. In the 1900s, *S* was 0.440 ± 0.081 for carbon and 0.386 ± 0.085 for nitrogen. The change of *S* between centuries was mostly driven by a shrinking BIC instead of a change in WIC; mean WIC for the 1800s for δ^13^C and δ^15^N was 0.457 ± 0.216 and 0.586 ± 0.323, respectively, and for the 1900s was 0.500 ± 0.183 and 0.516 ± 0.212. BIC showed a considerably larger difference, especially for nitrogen; BIC for 1800s carbon and nitrogen was 0.773 and 1.435, and for 1900s beluga these values dropped to 0.607 and 0.778.

**TABLE 3:** Individual specialization of beluga calculated from both nitrogen and carbon stable isotope ratios. Means ± standard deviations are reported. WIC, within individual component, BIC, between individual component, and S is the specialization index (lower values mean more specialized). Only one BIC is calculated per century, thus no standard deviation is reported.

		1800s	1900s
Carbon	WIC	0.457 ± 0.216	0.500 ± 0.183
	BIC	0.773	0.607
	S	0.355 ± 0.122	0.440 ± 0.081
Nitrogen	WIC	0.586 ± 0.323	0.516 ± 0.212
	BIC	1.435	0.778
	S	0.277 ± 0.103	0.386 ± 0.085

## 4 DISCUSSION

The samples analyzed here suggest that at the community level, Pacific Arctic beluga whales occupied a broader trophic niche and demonstrated higher individual specialization in the 1800s than in the 1900s. Every Layman metric calculated was greater for 1800s beluga than for those from the 1900s. Why there may have been a compression in the trophic niche width of Pacific Arctic beluga, a higher degree of dietary overlap among individuals, and lower degree of individual specialization from the 1800s to 1900s is difficult to assess, yet several plausible scenarios arise.

The first is the possibility of this niche compression being the result of our sampling design and due to the larger geographic sampling location of 1800s teeth from museum collections, the slightly greater temporal range covered by the 1800s samples (53 years for 1800s vs 43 years for 1900s), our small sample size, the uneven inclusion of males and females, or differences in ages of beluga between the two time periods. In regards to the uneven spatial and temporal coverage of data, results from our pairwise comparison analysis suggest that variation due to the total geographic and temporal scope of our teeth between sampled GLGs on ratios of δ^13^C & δ^15^N is minimal and similar, if not even less than, variation for GLGs collected from the same time and place. In fact, when visually inspecting δ^13^C–δ^15^N isospace for whales from the wider geographic range of the 1800s, the two most dissimilar individuals to each other were both collected from the same area, Norton Sound, AK (the Pastolik and Saint Michael Island individuals), while there was a strong overlap between one of these Norton Sound individuals and the individual from the Beaufort Sea (the largest range of latitudes present; Figure 2). Additionally, the larger trophic niche width of 1800s beluga arises despite this group only having half the sample size of the 1900s.

If we assume contemporary beluga stocks in the Pacific Arctic as we understand them today, both centuries would likely include individuals from two to three stocks each. It is worth noting that population dynamics of beluga from Kotzebue Sound are not well understood; it is believed that prior to approximately 1984, a unique Kotzebue Sound stock occurred in these waters that were genetically distinct from other Pacific Arctic stocks before a sharp population decline occurred and the sound became frequented by individuals from other Pacific Arctic stocks (based on indigenous knowledge of beluga movement patterns and genetic analysis; Castellote et al., 2022; Lucier & Vanstone, 1995; O'Corry‐Crowe et al., 2021). Teeth included in our study from Kotzebue Sound include data from 1983 at the very latest, conveniently timed prior to the mysterious population decline, and so likely belonged to beluga from the Kotzebue Sound stock only. Thus, despite collection locations for 1800s beluga covering a wider geographic area than the 1900s, we still might expect a similar level of stock diversity included in each temporal group. It is also worth noting the individuals with collection locations of Point Hope, AK, and northwards most likely belong to the Chukchi and Beaufort Sea stocks. These beluga undergo some of the longest migrations of all Pacific Arctic stocks, and as a result, might be expected to have larger standard ellipse areas due to the inclusion of prey items from drastically different summer and winter areas. Yet the individual with the most northern collection location of Nuvuk (Point Barrow; catalog ID 305071 from the 1800s group) had the smallest SEA of all included whales, regardless of century (Figure 3). Of the remaining 1900s beluga, there did not appear to be any patterns of SEA between individuals collected from Point Hope and Point Lay versus those from Kotzebue Sound (Figure 3). Thus, despite likely having considerably larger home ranges, the northernmost beluga examined in our limited set of specimens did not necessarily show a more generalist diet than the rest as indicated by stable isotope ratios.

We found female beluga to be significantly depleted in δ^15^N when compared to males, though we would not expect this to create major biases within temporal groups. For the 1900s beluga, there were approximately the same number of males and females. For the 1800s beluga, only one whale was known to be female while the rest were unknown, preventing any inferences from being made. However, this one female from the 1800s group had among the highest δ^15^N of all included whales (Figure 2; catalog ID 22207). Finally, age at death between belugas from the two time periods was not even; three individuals from the 1800s group were considerably younger at death than all beluga from the 1900s group. In female southern elephant seals, dietary specialization has been shown to increase with age (although not necessarily linearly) as individuals home in on foraging strategies (Hückstädt et al., 2012). Our analyses do not allow us to examine the degree of dietary specialization with age, although it should be noted that there were no discernable patterns in individual standard ellipse areas by age at death (Figure 3). Nonetheless, it is possible that uneven distributions of males and females, as well as the age at death of individuals, between both time periods could affect our results.

Our findings of a compressed niche width and higher levels of dietary generalization in 1900s beluga could also be explained by a multitude of scenarios concerning competition for resources, changes in beluga prey diversity and availability (ecological opportunity), large‐scale shifts in biological communities (e.g., invasion or northward expansion of novel species), or predation. Given the limits of relying exclusively on bulk stable isotope ratios of carbon and nitrogen taken from teeth of historic samples and uncertainties regarding the Pacific Arctic marine environment from as far back as the mid‐1800s, it is impossible to rule out any one of these scenarios in favor of another. As ratios of δ^13^C and δ^15^N are assimilated into the tissues of a consumer from its total diet during the period of tissue synthesis, any change in niche width of a consumer is ultimately due to a change in the diversity of prey consumed (Layman, Quattrochi, et al., 2007). This could be the result of consumers foraging on a larger or smaller subset of prey or an expansion or contraction in the trophic diversity of the food web as a whole; Layman, Quattrochi, et al. (2007) found that a less diverse set of basal resources was a principal driver in the collapse of the niche width of a top predator in fragmented aquatic ecosystems. While historical isotope data from the base of the food web are not available, future research could incorporate compound‐specific stable isotope analysis to allow further inferences regarding the occurrence, magnitude, or effect that a change in the baseline δ^13^C & δ^15^N of the Pacific Arctic marine environment may have had on the isotope ratios of our beluga teeth.

Factors such as prey availability or diversity, competition between and within species, and predation risk (increasingly an issue due to northward expansion of orcas) can independently and synergistically affect consumer trophic dynamics and would also presumably affect the degree of individual specialization among individuals (reviewed in Araújo et al., 2011). For example, competition for resources between and within species can either increase or decrease the trophic niche width of a consumer. Optimal foraging theory postulates that when intraspecific competition for a limited amount of resources is high, certain individuals in a population will be pressured to begin selecting for suboptimal prey types, thereby increasing the overall niche width at the population level and increasing dietary specialization and differentiation at the individual level (Kernaléguen et al., 2015; Ratcliffe et al., 2018; Tinker et al., 2012; Yurkowski et al., 2016). If the effects of intraspecific competition and prey availability as they relate to optimal foraging theory are at play here, beluga may have shown a compressed niche and higher degree of dietary overlap due to reduced competition for resources over time. In this hypothetical scenario, a release from high levels of intraspecific competition (either from less beluga or more prey) would facilitate more individuals of a population being able to select for higher quality prey and a subsequent decrease in population‐level niche width as well as a decrease in the degree of individual specialization.

Any changes in the Pacific Arctic marine food web of a sufficient magnitude to affect the isotopic profiles of beluga prey, or simply the addition or removal of prey species, will affect the isotopic ratios found in these beluga teeth. Warming and increasingly ice‐free areas of the Pacific Arctic are facilitating the northward expansion of certain prey species, such as capelin (*Mallotus villosus*; Grebmeier, 2012; Spies et al., 2019). Capelin have been expanding into both the Pacific and Atlantic sectors of the Arctic and are likely becoming an increasingly popular dietary component of beluga, especially females and younger individuals which have a stronger spatial overlap with capelin as they forage closer to shore (Choy et al., 2020; Gaston et al., 2003; Marcoux et al., 2012). Yurkowski et al. (2018) found that warming waters and northward range expansions of novel prey, in particular capelin, resulted in a dietary shift of Arctic marine predators (including beluga) in the Cumberland Sound between 1990 and 2012 and a subsequent decrease in the trophic diversity of that predator community. In their study, an assemblage of beluga, ringed seals (*Pusa hispida*), Greenland halibut (*Reinhardtius hippoglossoides*), and Arctic char (*Salvelinus alpinus*) shifted from a higher to lower level of trophic diversity and a higher level of trophic redundancy as indicated by the same six Layman metrics and standard ellipse areas calculated here (Yurkowski et al., 2018). The authors argued that this decrease in trophic diversity was primarily driven by a reduction in the diversity of basal resources (similar to Layman, Quattrochi, et al., 2007) due to predators eating novel prey (e.g., capelin) and switching largely to a pelagic energy pathway. Capelin have been documented as far north as the Beaufort Sea as early as the 1960s; this encroachment is quite recent relative to the timeline covered by our 1900s samples and may not be as relevant to our results (Choy et al., 2020; McNicholl et al., 2017). Still, all but one beluga from our 1900s community had a date of death of approximately 1977–1983 so it is possible that the encroachment of capelin, and/or similarly novel species moving north as oceans warm, could have been at play here. Finally, the Pacific Arctic experiences strong, decadal‐scale regime shifts due largely to climatic drivers (e.g., Pacific Decadal Oscillation, North Pacific Gyre Oscillation, and El Niño‐Southern Oscillation, among others), where species assemblages are significantly altered (Brodeur et al., 2008; Conners et al., 2002; Ebbesmeyer et al., 1991; Hare & Mantua, 2000; Litzow et al., 2014; Niebauer, 1998). These regime shifts likely have a profound effect on the biology of marine mammal predators which may be reflected in isotope ratios (see York et al., 2008). Additionally, numerous studies have detected long‐term changes in primary production and carrying capacity in the region using time series data from marine mammal tissues (Newsome, Etnier, et al., 2007; Schell, 2000).

While recent studies show significant evidence for contemporary shifts in food webs and predator foraging behavior (Choy et al., 2020; Marcoux et al., 2012; Watt et al., 2016), the data presented here suggest that diet shifts may also have occurred prior to the most significant climate‐driven changes in the region. It seems possible that over the 138‐year timeframe of the present study, there may have been numerous shifts in the prey landscape for beluga that would have resulted in isotopic shifts or changes in niche width. The scale and nature of this trophic shift may provide helpful context for contemporary observations that aim to understand the increasing impact of climate change on beluga whale diet, health, and behavior. On one hand, the shifts documented here suggest beluga have already been quite plastic in their trophic behavior, both in terms of individual specialization and community niche breadth. While that suggests they may continue to adapt in the face of current climate‐driven changes, the compression of niche width may also indicate an increasingly limited foraging landscape for the species which may decrease resiliency. As one of just a few endemic Arctic cetaceans and a culturally and nutritionally significant resource for Alaska Natives, further work on the foraging behavior of beluga and their Pacific Arctic food web should be prioritized.

## AUTHOR CONTRIBUTIONS

**Devin C Fraleigh:** Conceptualization (equal); data curation (equal); formal analysis (lead); methodology (equal); visualization (lead); writing – original draft (lead); writing – review and editing (equal). **Frederick I Archer:** Formal analysis (equal); writing – review and editing (supporting). **Amanda S Williard:** Conceptualization (supporting); writing – original draft (supporting); writing – review and editing (supporting). **Luis A Hückstädt:** Conceptualization (supporting); writing – original draft (supporting); writing – review and editing (supporting). **Alyson H Fleming:** Conceptualization (equal); data curation (equal); formal analysis (equal); funding acquisition (lead); methodology (equal); project administration (lead); resources (equal); writing – original draft (supporting); writing – review and editing (supporting).

## FUNDING INFORMATION

Smithsonian Institution's James Smithson Fellowship and National Science Foundation grant #1927742.

## CONFLICT OF INTEREST STATEMENT

The authors declare no conflicts of interest.

## Data Availability

The data that support the findings of this study are openly available in Dryad at https://doi.org/10.5061/dryad.3bk3j9kqp.
